# Supplementary material for: Chromatin accessibility dynamics reveal novel functional enhancers in C. elegans
Source: Genome Res. 2017 Dec;27(12):2096–107. doi: 10.1101/gr.226233.117 (PMC5741055; doi:10.1101/gr.226233.117)
Supplement: Supplemental Material [file supp_27_12_2096__index.html]

Chromatin accessibility dynamics reveal novel functional enhancers in C. elegans — Supplemental Material 

# Chromatin accessibility dynamics reveal novel functional enhancers in *C. elegans*

## Supplemental Material

undefined

- Supplemental\_Material.pdf
- Supplemental\_Table\_S1.xlsx
- Supplemental\_Table\_S2.xlsx
- Supplemental\_Table\_S3.xlsx
- Supplemental\_Table\_S4.xlsx
- Supplemental\_Table\_S5.xlsx
- Supplemental\_Table\_S6.xlsx
- Supplemental\_Table\_S7.xlsx
- Supplemental\_Table\_S8.xlsx
- Supplemental\_Table\_S9.xlsx
- Supplemental\_Table\_S10.xlsx
- Supplemental\_Table\_S11.xlsx
- Supplemental\_Table\_S12.xlsx
- Supplemental\_Table\_S13.xlsx
- Supplemental\_Table\_S14.xlsx
- Supplemental\_Extended\_Protocol.docx
- Supplemental\_CelegansATACseq-master.zip
- Supplemental\_Extended\_Protocol.xls
