# Supplementary material for: Chromatin accessibility dynamics reveal novel functional enhancers in C. elegans
Source: Genome Res. 2017 Dec;27(12):2096–107. doi: 10.1101/gr.226233.117 (PMC5741055; doi:10.1101/gr.226233.117)
Supplement: Supplemental Material [file supp_gr.226233.117_Supplemental_Material.pdf]

Supplemental Materials

for

Chromatin accessibility dynamics reveal novel functional enhancers in *C. elegans*

Aaron C. Daugherty[1,7], Robin W. Yeo[1,7], Jason D. Buenrostro[1,2,3], William J. Greenleaf[1,4], Anshul Kundaje[1,5], Anne Brunet[1,6*]

[1] Department of Genetics, Stanford University, Stanford CA 94305, USA

[2] Present address: Broad Institute of MIT and Harvard, Harvard University, Cambridge, MA 02142, USA

[3] Present position: Harvard Society of Fellows, Harvard University, Cambridge, MA 02138, USA

[4] Department of Applied Physics, Stanford University, Stanford, CA 94305, USA

[5] Department of Computer Science, Stanford University, Stanford, CA 94305, USA

[6] Glenn Laboratories for the Biology of Aging, Stanford University, Stanford CA 94305, USA

[7] These authors contributed equally to this work

* Corresponding author. Email: anne.brunet@stanford.edu

Table of Contents

Supplemental Figures

- Supplemental Figure 1 Page 3-4
- Supplemental Figure 2 Page 5-6
- Supplemental Figure 3 Page 7-8
- Supplemental Figure 4 Page 9-10
- Supplemental Figure 5 Page 11-12
- Supplemental Figure 6 Page 13-14
- Supplemental Figure 7 Page 15-16

Supplemental Tables Page 17

Supplemental Methods Page 18-38

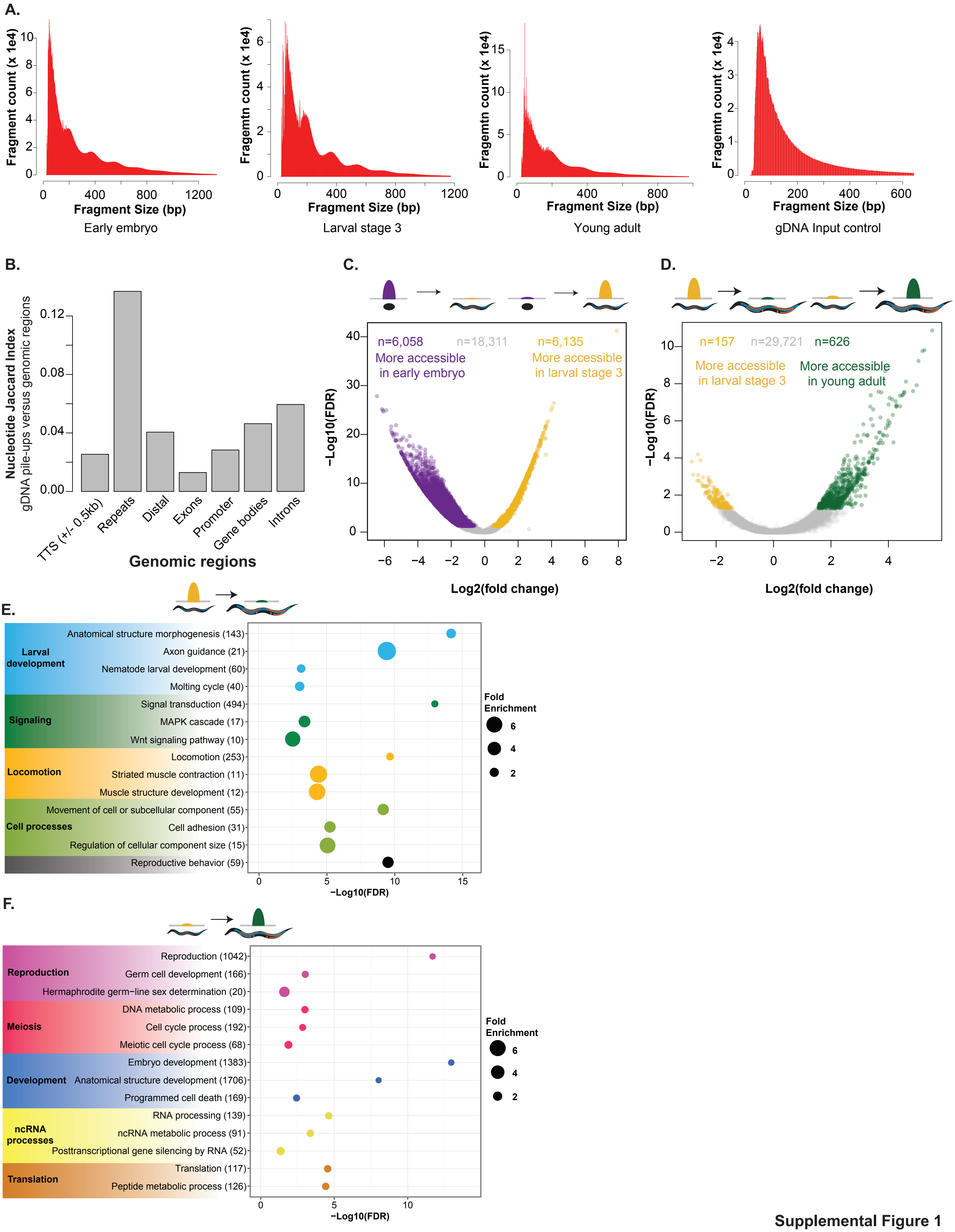

Supplemental Figure 1

Supplemental Figure 1. (*A*) Histograms of fragment size for representative experimental replicates (first three panels from the left) show consistent approximately 147bp periodicity, likely corresponding to nucleosome-protected fragments. This periodicity is not present in the purified genomic DNA (gDNA) input control (far right panel). (*B*) Read pile-ups in genomic DNA input control overlap annotated repeats. The single base pair Jaccard index (a measure of nucleotide overlap) was calculated for the called gDNA read pile-ups versus genomic locations (downloaded from the UCSC Genome Browser). (*C,D*) Significantly differential consensus ATAC-seq peaks between early embryo (embryo) and larval stage 3 (L3) (C), and larval stage 3 (L3) and young adult (adult) (*D*); FDR < 0.05). (*E,F*) Genes that lose accessibility between L3 and adult are enriched for larval development functions (E), while genes that gain accessibility are enriched for adult-related functions (F); all calculations and genes lists are from GOrilla and the number of genes contributing to the enrichment of each term are listed in parentheses.

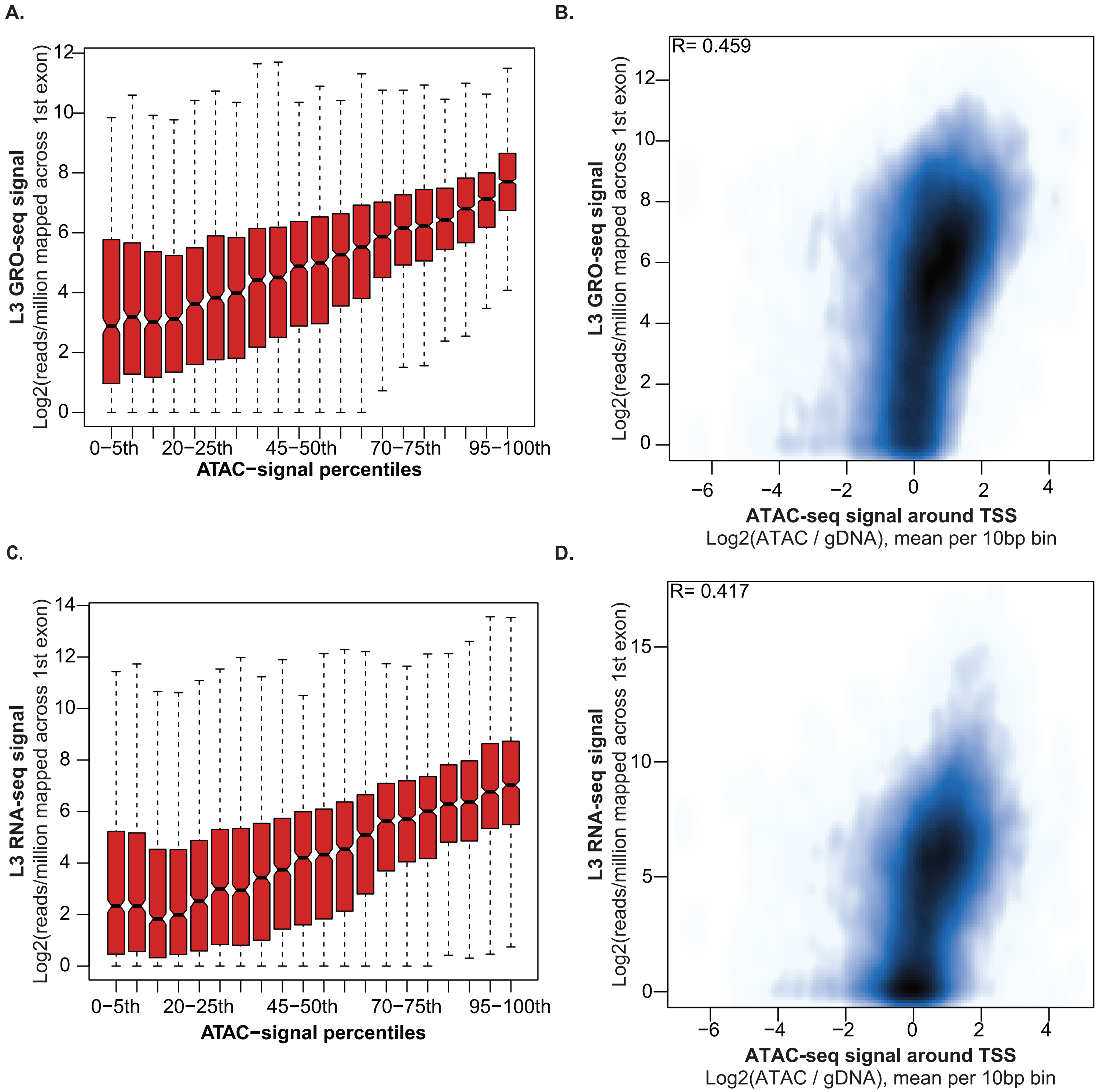

Supplemental Figure 2

Supplemental Figure 2. (*A*) Interquartile bar plots illustrating transcriptional activity with increasing ATAC-seq signal (binned as 5 percentile ranges). For each gene, the ATAC-seq signal (+/- 1kb around the TSS) and nascent transcription (RPKM across RefSeq first exons) was calculated using L3 ATAC-seq data and publicly available L3 GRO-seq data. (*B*) Smoothed scatterplot illustrating the same relationship between TSS accessibility and nascent transcription (Pearson r is shown). (*C, D*) Interquartile bar plot and smoothed scatterplot illustrating the relationship between TSS accessibility and transcriptional activity using publicly available RNA-seq data at the L3 stage.

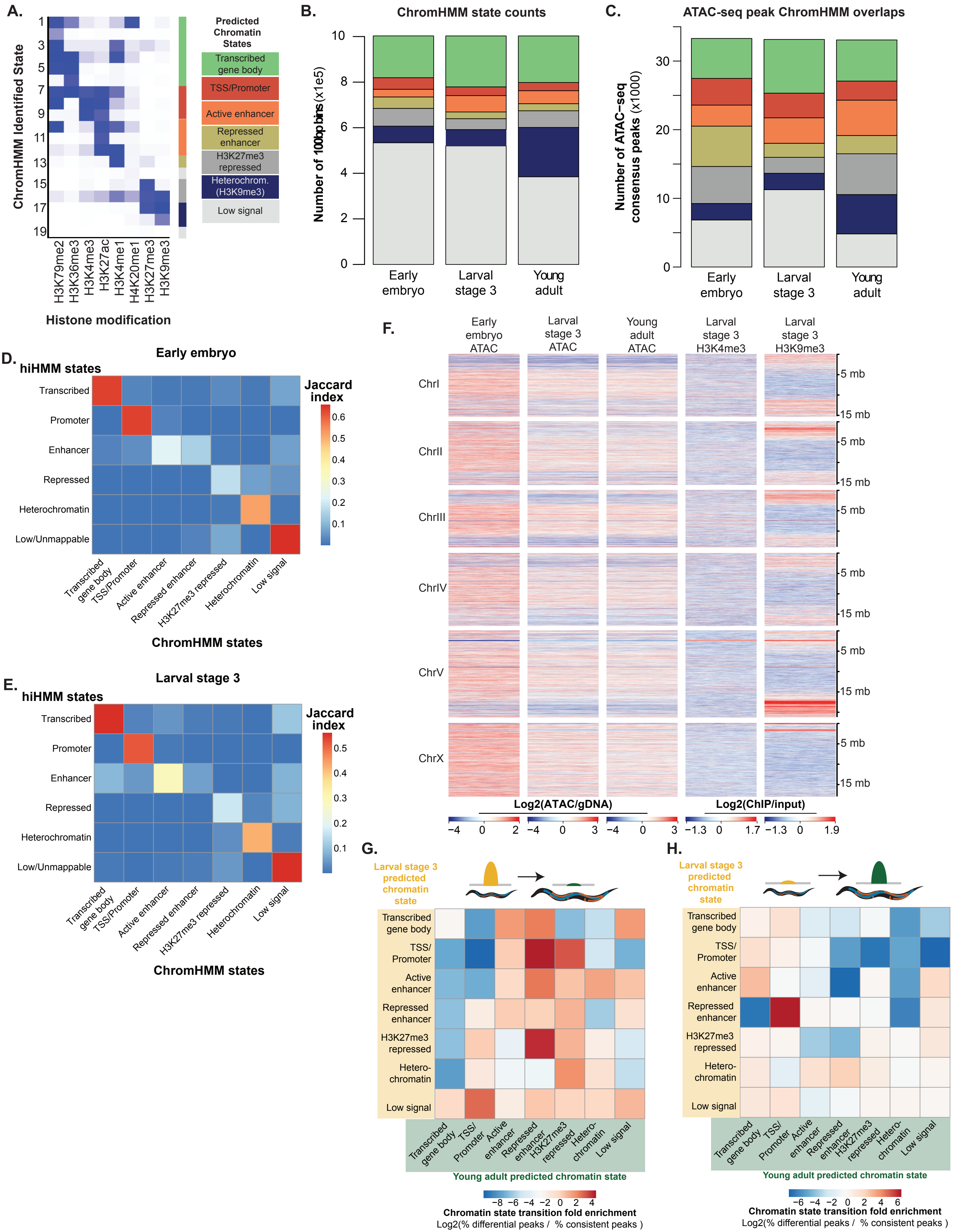

Supplemental Figure 3

Supplemental Figure 3. (*A*) Heatmap of emission parameters from ChromHMM show enrichment of the histone modifications in each ChromHMM-predicted chromatin states as well as the 7 core chromatin states. Each of the 19 original states was assigned to a core chromatin state using canonical associations of histone modifications. (*B*) The number of 100bp bins (the resolution used for building the ChromHMM model) found in each predicted state for each of the three stages. (*C*) The number of ATAC-seq peaks which overlapped by at least half their length in each stage-specific ChromHMM predicted state. (*D,E*) ChromHMM predicted chromatin states closely resemble predictions made by (Ho et al. 2014) using hiHMM a method similar to ChromHMM. For both early embryo (D) and larval stage 3 (E) the Jaccard index (a measure of nucleotide overlap) for each of the 7 ChromHMM-predicted states was calculated for each of the 6 chromatin states predicted by Ho et al. 2014 using hiHMM. (*F*) ATAC-seq signal is highest in the gene-dense center of *C. elegans* chromosomes and notably anti-correlated with heterochromatin-associated H3K9me3 ChIP-seq. (*G,H*) Decreases in accessibility in ATAC-seq peaks are enriched for transitions from active regulatory to inactive chromatin states (G), while increases in accessibility are enriched for transitions from inactive chromatin states to active regulatory states (H).

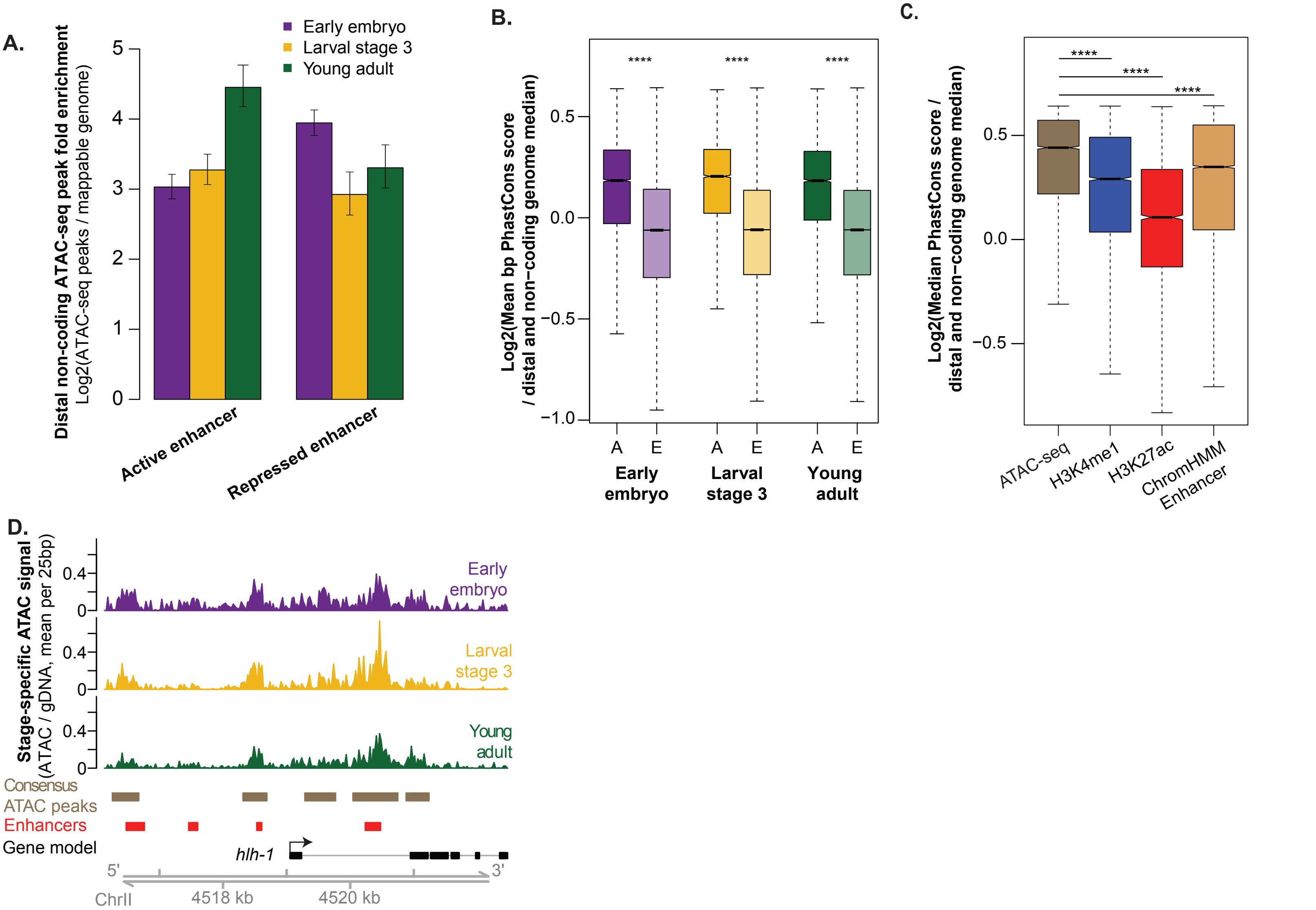

Supplemental Figure 4

Supplemental Figure 4. (*A*) Distal non-coding ATAC-seq peaks are enriched for ChromHMM-predicted enhancer chromatin states. Significance was assessed using 10,000 bootstrap iterations and error bars represent 95% confidence intervals (all enrichments $p < 1e-4$). (*B*) The median single base pair conservation score for every stage-specific distal non-coding ATAC-seq peak was normalized to the genome-wide distal non-coding median (A: Actual, darker hues) and compared to expected scores (E: Expected, light hues) derived from randomizing peak locations across the distal non-coding genome (*** $p < 1e-323$, Kolmogorov-Smirnov (KS) test). (*C*) Consensus distal non-coding ATAC-seq peaks are more highly conserved than distal non-coding histone modification peaks often associated with enhancers. Pooled H3K4me1 and H3K27ac ChIP-seq peaks are from all three stages. ChromHMM enhancers are regions predicted to be in an active or poised enhancer state in any of the three stages assayed. *** $p < 1e-323$, KS test. (*D*) Consensus ATAC-seq peaks overlap experimentally defined enhancers near the MyoD homolog, *hlh-1*. Functionally defined enhancers (red) were from (Lei et al. 2009).

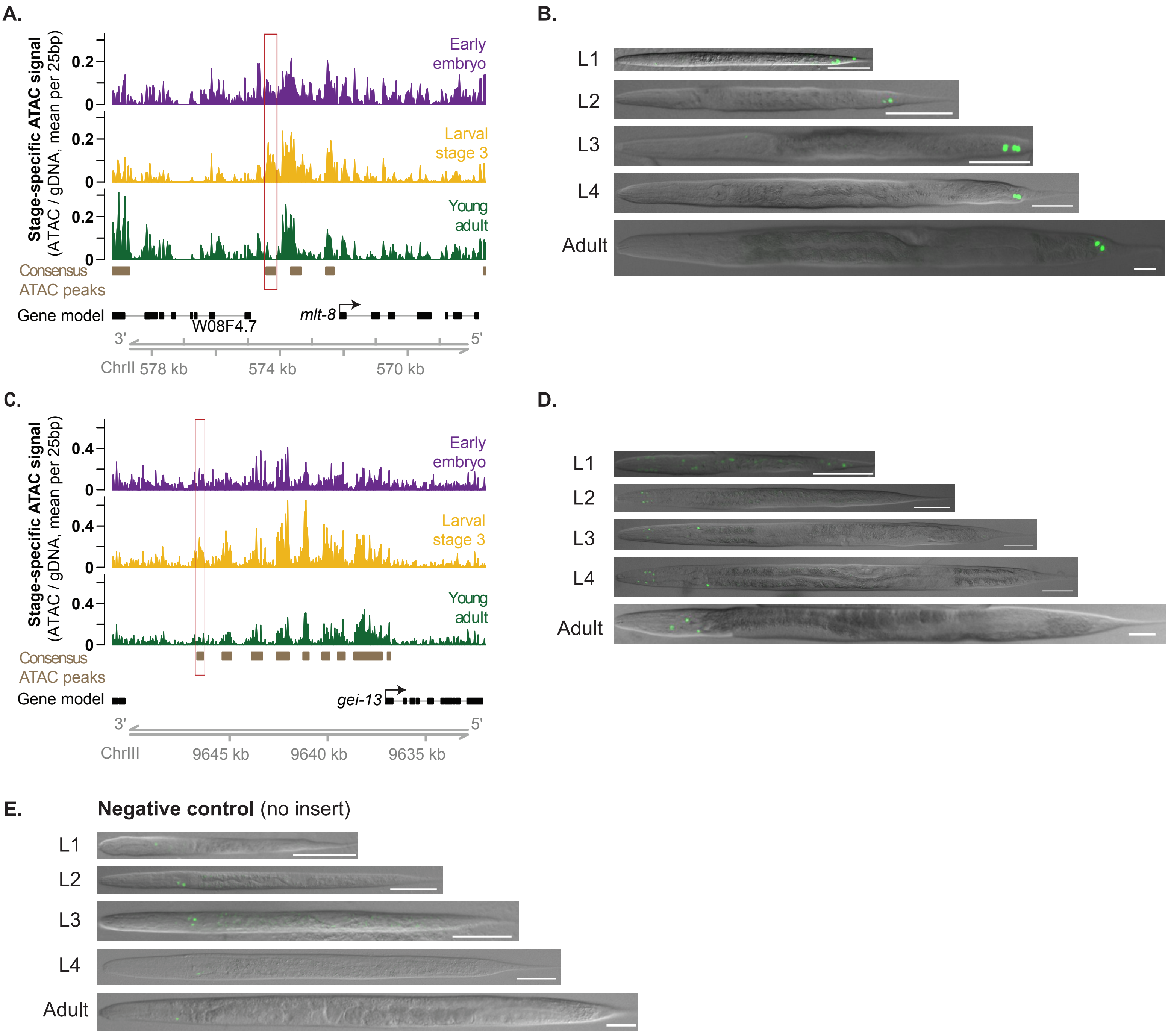
A.
Stage-specific ATAC signal
(ATAC / gDNA, mean per 25bp)
0.2
0
Early
embryo
Larval
stage 3
Young
adult
Consensus
ATAC peaks
Gene model
W08F4.7
mlt-8
3'
5'
ChrII 578 kb
574 kb
570 kb
B.
L1
L2
L3
L4
Adult
C.
0.4
gei-13
ChrIII
9645 kb
9640 kb
9635 kb
D.
E.
Negative control (no insert)

Supplemental Figure 5. (*A,C*) ATAC-seq signal near *mlt*-8 and *gei-13* including the regions tested for enhancer activity (boxed in red). Note both plots are in reverse orientation for consistency with Figure 4. (*B,D,E*) Representative images of staged *C. elegans* transgenic lines for *mlt-8* (B), *gei-13* (D) and no-insert negative controls (E). The scale bar is 50 µm, and all images were straightened with ImageJ and are greyscale images with florescence overlaid.

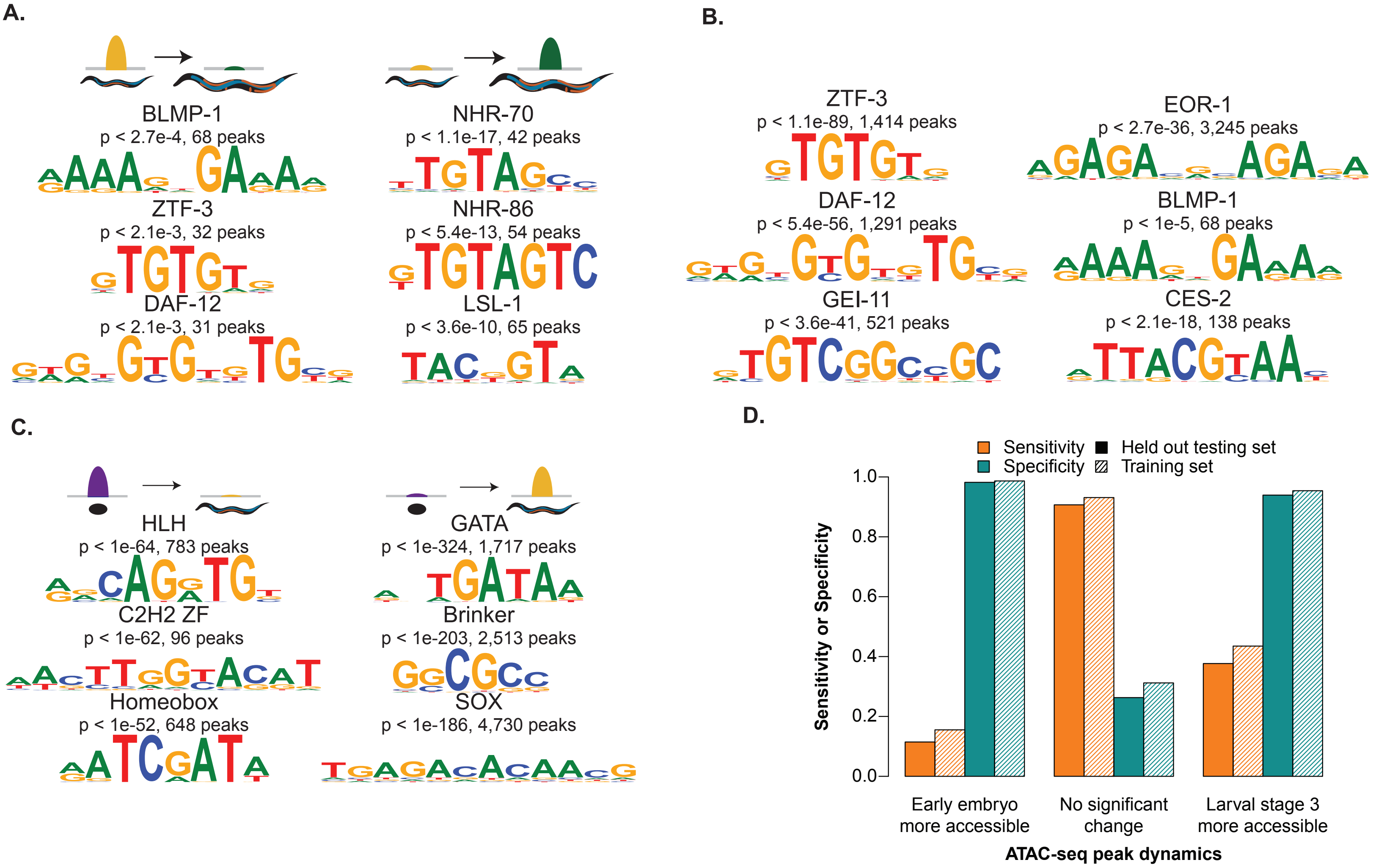

Supplemental Figure 6

Supplemental Figure 6. (*A*) ATAC-seq peaks which decreased (left) or increased (right) accessibility between L3 and young adult are enriched for previously identified transcription factor binding motifs; p-values are Benjamini-Hochberg corrected for multiple hypothesis testing. (*B*) Enrichment of experimentally defined *C. elegans* transcription factor motifs in distal non-coding ATAC-seq peaks; p-values are Benjamini-Hochberg corrected for multiple hypothesis testing. (*C*) *de novo* motifs identified in peaks which were decreased (left) or increased (right) between embryo and L3. Motifs were discovered using Homer and a background set of all unchanging ATAC-seq peaks. (*D*) Evaluations of predictions of chromatin accessibility changes between L3 and early embryo. The sensitivity (True Positive Rate) and specificity (True Negative Rate) for both testing and training sets of peaks were determined for each dynamic ATAC-seq peak type.

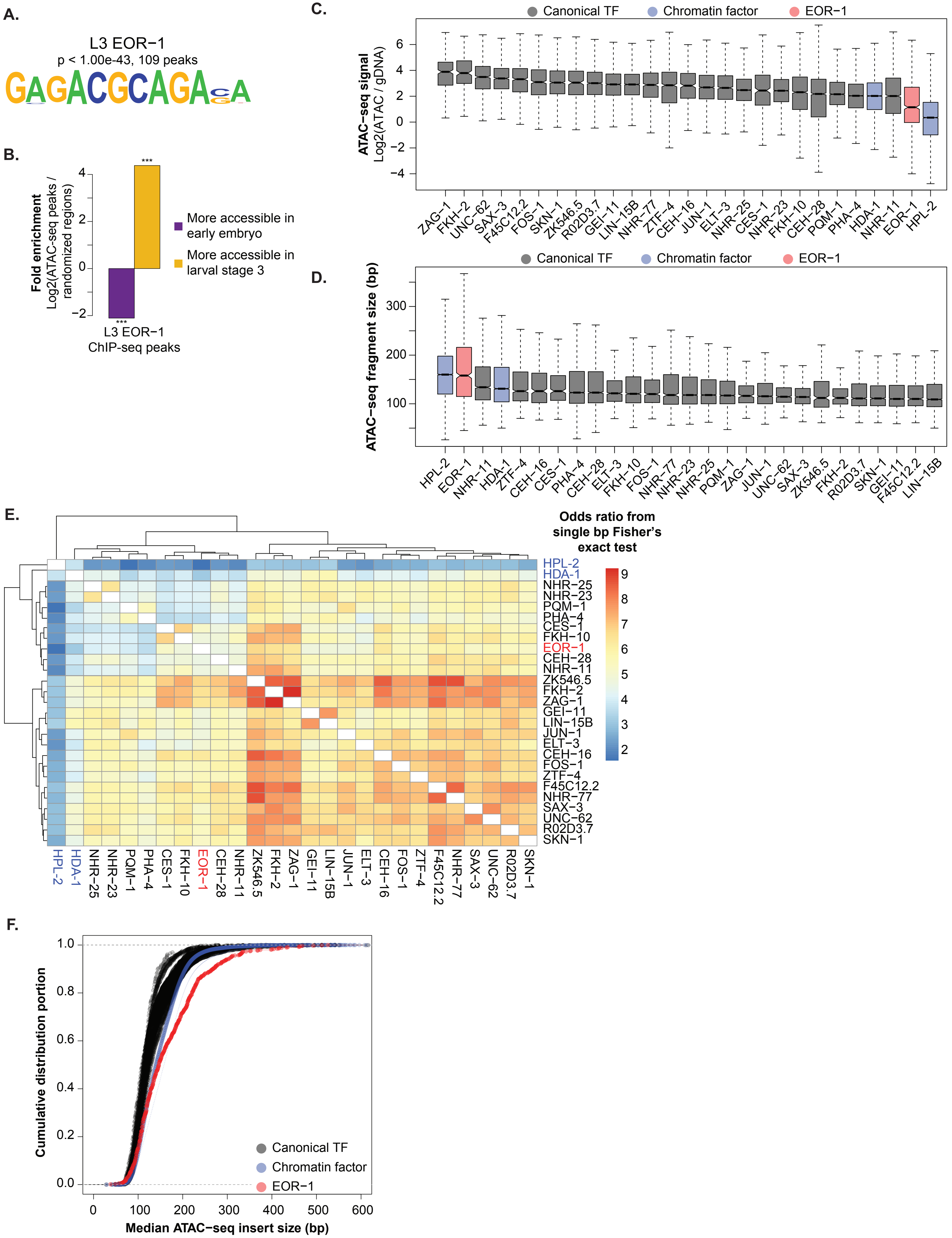

Supplemental Figure 7

Supplemental Figure 7. (*A*) The most significantly enriched *de novo* motif in the L3 EOR-1 ChIP-seq peaks. (*B*) L3 EOR-1 ChIP-seq peaks are significantly enriched for ATAC-seq peaks that increase in accessibility between early embryo and L3. Fold enrichment and significance was calculated by comparison to a null distribution of 10,000 iterations. (*C,D*) L3 EOR-1 ChIP-seq peak summits are less accessible than other L3 canonical TF and chromatin factor ChIP-seq peak summits. For each TF ChIP-seq, ATAC-seq signal (C) and fragment size (D) within the midpoint (+/- 50 bp) of every peak is reported. (*E*) Heatmap illustrating the co-localization of L3 ChIP-seq peaks overlapping the peaks of other factors. Odds Ratio calculated from Bedtools' implementation of Fisher's exact test, which uses the entire genome as background. *(F)* The cumulative distribution functions for the median L3 ATAC-seq fragment size at the midpoint (+/- 50 bp) of L3 ChIP-seq peak summits represented in Figure 4E.

For all supplemental tables, please see corresponding excel spreadsheet Supplemental_Table_SX.xlsx

Supplemental Table 1. Number of mapped reads for the three biological ATAC-seq replicates at early embryo, larval stage 3 and young adult as well as the genomic DNA input control.

Supplemental Table 2. Number of high-confidence peaks called at each stage.

Supplemental Table 3. Detailed annotations for all consensus ATAC-seq peaks, including genomic position, nearest gene, genomic identity, differential accessibility across the 3 developmental stages, and predicted ChromHMM state.

Supplemental Table 4. All Gene Ontology (GO) - Biologic Process (BP) terms enriched in genes with more accessibility in early embryo than larval stage 3.

Supplemental Table 5. All GO-BP terms enriched in genes with more accessibility in larval stage 3 than early embryo.

Supplemental Table 6. All GO-BP terms enriched in genes with more accessibility in than larval stage 3 than young adult.

Supplemental Table 7. All GO-BP terms enriched in genes with more accessibility in young adult than larval stage 3.

Supplemental Table 8. Details of modENCODE histone modification ChIP-seq datasets used.

Supplemental Table 9. Genome-wide ChromHMM predictions at early embryo.

Supplemental Table 10. Genome-wide ChromHMM predictions at larval stage 3.

Supplemental Table 11. Genome-wide ChromHMM predictions at young adult.

Supplemental Table 12. Putative regulatory regions tested in functional enhancer assay.

Supplemental Table 13. Primer sequences used for cloning putative regulatory regions for functional enhancer assay.

Supplemental Table 14. Strain information for transcription factor ChIP-seq datasets used in transcription factor analyses.

Supplemental Methods

Maintenance and strains

All *C. elegans* lines were maintained on Nematode Growth Medium agar plates at 20°C using *Escherichia coli* OP50.1 as a food source. Wild-type (N2) worms were provided by Dr. Man-Wah Tan.

Plasmids for enhancer screen

The minimal *Ppes-10*::4xSV40NoLS::GFP::*let-858* 3-UTR plasmid (pL4051) used for enhancer screening was a gift from Andrew Fire (Addgene plasmid #1629). The pRF4 co-injection marker plasmid *Prol-6::rol-6(su1006)* was a gift from Stuart Kim's lab.

Enhancer GFP plasmid construction

For the enhancer screen, each putative regulatory region was cloned in the pL4051 plasmid, upstream of a minimal promoter (*pes-10*) driving expression of a *C. elegans* intron- and photo-stability-optimized GFP containing an N-terminal nucleolar localization signal (NoLS).

Putative regulatory regions were chosen by selecting ATAC-seq peaks that exhibited the largest differential accessibility between two stages and that were at least 1kb from a transcription start site. Flanking negative control regions were chosen by selecting regions within 2kb of the putative regulatory regions that were not in peaks of accessibility. Primers were designed to amplify each region as well as 50-500bp flanking either side (Supplemental Table 8). The fragments were amplified from genomic DNA extracted from N2 worms using NEBNext High-Fidelity and cloned into the pL4051 plasmid. Cloned PCR fragments were sequence-verified.

Transgenesis

Wildtype N2 day 1 adults were microinjected following the standard protocol (Mello and Fire 1995) with a mix containing 75 ng/µl of the respective enhancer-GFP reporter constructs and 75 ng/µl of pRF4, a *rol-6* co-injection marker.

Enhancer screen in *C. elegans*

Stable extrachromosomal transgenic lines for putative enhancer regions determined by ATAC-seq peaks, negative control regions (regions flanking ATAC-seq peaks), or the no-insert control were generated. For each transgenic line, mixed-staged worms were screened for GFP signal distinct from the no-insert control background signal, which is 1-2 nuclei near the pharynx in all larval and adult stages (Supplemental Fig. 5E). To quantify the consistency of GFP expression, all lines (including the negative control lines) (Table 1) were scored for GFP expression in a blinded manner.

For those regulatory regions that displayed a consistent GFP expression pattern in transgenic worms, we also generated 2-5 stable transgenic lines in which the region was inverted from its endogenous orientation relative to the putative TSS (as defined by the UCSC Genome Browser). These transgenic lines were assessed in the same manner as described above. In most cases, the closest downstream TSS was assigned as the putative TSS. However, in cases where a TSS was not present on either side of the region (e.g. *nhr-25*), we preferentially selected the closest TSS while also considering the GFP spatiotemporal activity we observed and the canonical functions of the neighboring genes.

Fluorescence microscopy

Transgenic worms were synchronized via a timed egg lay and prepared for live imaging at the indicated stages. Larval and adult worms were washed off plates in 100 mM levamisole (MP Biomedicals), resuspended in M9, allowed to settle, and washed two additional times with levamisole. Larval and adult worms were washed for a minimum of 30 minutes to clear bacteria from the gut. Worms were transferred to 2% agarose pads and imaged using a Zeiss AxioSkop 2 Plus at 20x magnification for larvae or 10x magnification for adults. Images were acquired using an AxioCam MRc camera with AxioVision 4.7 software. Exposure times were kept constant for all stages of each strain. DIC and GFP images were merged and worm bodies were straightened in FIJI. One representative image is shown per stage with 50 μm scale bars.

Collection of timed samples for ATAC-seq

For ATAC-seq, three sets of completely independent biological replicates (i.e. performed at different times) were prepared in the following manner. To synchronize the parents of each sample, well-fed mixed stage N2 worms from one 10cm or three 6cm plate(s) (approximately 500 worms) were treated with 10% bleach for 4 min to isolate early embryos (as described in Wormbook). The resulting embryos (approximately 800) were grown to adulthood on 2-3 10cm plates. As soon as the worms reached adulthood, they were placed on fresh 10cm plates at a density of approximately 100 worms per plate for synchronized egg-laying. After 45 min, the adults were removed, and the plates were returned to 20°C to allow the embryos to grow to the desired stages.

To collect the embryos, the egg-laying adults were collected, and approximately 50 μl of packed adults were washed once with M9 medium and bleached to yield early embryos as

described in Wormbook. Following the last wash after bleaching, the embryos were left in 100 μl of M9 medium, and the tubes flash frozen in liquid nitrogen and kept at -80°C. The parents were also flash frozen and used for genomic DNA controls.

To collect larval stage 3 (L3) worms, plates were repeatedly checked after egg-laying to ensure no parents had remained. Then, 36 hr after the start of the egg lay, L3 animals were collected from 2 10cm plates. Finally, to collect young adult worms, 57 hr post egg lay one of the remaining plates was checked every 10 min to ensure that the majority of the plate was composed of young adult animals with no more than 1-2 eggs. At that point, young adult animals were harvested by washing the plate with M9 medium and collecting the samples in a 15 ml tube. Immediately after collection, all worm samples were centrifuged gently at 200 g and washed twice with 10 ml of M9. All extra medium was removed and the samples were flash frozen in liquid nitrogen and stored at -80°C.

Nuclei purification and ATAC-seq of samples

Nuclei were purified from frozen samples as previously described (Haenni et al. 2012). Briefly, flash frozen samples were thawed on ice, and then mixed with 150 μl of 2X nuclei purification buffer (20mM Hepes pH 7.6, 20mM KCl, 3mM $MgCl_2$, 2mM EGTA, 0.5 M Sucrose, 0.05% Triton, 1mM DTT, 0.05M NaF, 40mM β-glycerophosphate, 2mM Na_3VO_4). All the following steps were performed at 4°C. The samples were transferred to a pre-chilled Wheaton stainless-steel homogenizer (Wheaton catalog number 357572) and were homogenized with 3 plunger strokes. The resulting samples were centrifuged at 200 g for 1 min to remove worm debris. The supernatant was transferred to a fresh tube being careful to not include any pellet debris; this resulted in approximately 50 μl of nuclei purification buffer being left with the pellet. The

remaining supernatant and pellet were further resuspended in 150 μl of 2X nuclei purification buffer and homogenized as described above. The process was repeated until no visible pieces of worm remained (approximately 5 times). The pooled supernatants were centrifuged at 200 g for 1 min to remove any remaining worm debris. Finally, the nuclei were pelleted at 1000 g for 10 min. As this step does not result in a visible pellet, care was taken to not disturb the spot where the pellet should be while completely removing the supernatant.

The purified nuclei were immediately used for the ATAC-seq protocol (Buenrostro et al. 2013). Briefly, the nuclei were resuspended in 47.5 μl of Nextera Tagmentation buffer (Nextera DNA Sample Preparation Kit) and incubated with 2.5 μl of the Tn5 transposase at 37°C for 30 min. Resulting DNA fragments were purified using a miniElute column (Qiagen) and amplified by NEBNext High-Fidelity PCR Master Mix in a total volume of 50 μl. The thermocycling protocol for this reaction was 72°C for 5 min, 98°C for 30 s and 5 cycles of 98°C for 10 s, 63°C for 30 s and 72°C for 1 min. Every sample shared the Adapter1 primer, and had a unique barcoded Adapter2 primer (Supplemental Table 8). To ensure over-amplification did not occur, after the initial 5 cycles, the number of remaining cycles required was estimated for each sample using qPCR (BioRad CFX96 Real-Time System). To do so, a similar reaction to the above was set up (NEBNext, Adapter1 and a single Adapter2), with the addition of SYBRGreen, and using 5 μl of the previous PCR as template. The final volume was 15 μl, and the thermocyling was as above except that the initial incubation step was 98°C for 30 seconds, and 40 cycles were performed. The number of additional cycles was determined to be the number it took for the qPCR to reach one-third maximal fluorescence. The original PCR was then resumed and each sample cycled as necessary. Following amplification, the samples were purified using QIAquick

columns (Qiagen), and library quality was verified on an Agilent bioanalyzer. Sequencing was performed using 101 bp paired-end sequencing on an Illumina Hi-seq 2000.

Isolation and ATAC of gDNA controls

To generate an input control for ATAC-seq, approximately 100 μl of packed adults was thawed and embryos were collected as described above to avoid any bacterial contamination. Next, genomic DNA was isolated by proteinase K treatment (0.2 mg/ml, 55°C for 3 hr before heat killing the proteinase K at 95°C for 25 min), then RNase treatment (1.25 mg/ml, 37°C for 30 min). gDNA was purified by double phenol/chloroform/isoamyl alcohol (25:24:1, pH 8.0) extraction, with an additional chloroform extraction, and precipitated with ethanol and NaOAc (3mM, pH 5.5), and resuspended in 50 μl TE (10mM Tris-HCl pH7.5, 1mM EDTA). The gDNA was quantified using a Qubit, and 10 ng used for a standard ATAC-seq protocol as described above.

Genomic analysis

For all analyses, the ce10/WS220 version of the *C. elegans* genome (Rosenbloom et al. 2015) was used. Base version of Perl (v5.16.3), Python (v2.7.9) and R (R Core Team 2013) as well as Samtools (v0.1.19-44428cd) and Bedtools (v 2.21.0) (Quinlan and Hall 2010) were used throughout, unless noted otherwise.

Gene definitions

RefSeq gene definitions were downloaded from UCSC Genome Browser and the transcription start sites (TSSs) were augmented with experimentally defined TSSs (Chen et al. 2013), when

possible. This gene definition (RefSeq genes with experimentally defined TSSs (Chen et al. 2013)) was used for all genomic analyses in the manuscript. For specific examples, including the choice of distal non-coding regions to test by transgenesis, TSSs from UCSC genome browser were used (for ease of visualization).

Mappable regions

The mappable genome was defined as those regions that single end 100bp reads could be mapped. This was accomplished using HotSpot (John et al. 2011).

ATAC-seq read alignment and quality filtering

The nine experimental ATAC-seq libraries, as well as an input control (ATAC-seq on purified genomic DNA) were sequenced to a median depth of over 17 million unique, high-quality mapping reads per sample (Supplemental Table 1). Sequencing adaptors were trimmed using a custom script that aligns the 5' ends of the forward read and reverse complement of the reverse read, and then removes any aligning sequence (minimum length 3). Subsequently, all reads were aligned to the ce10 version of the *C. elegans* genome, including mitochondrial sequence, with bowtie2 (Langmead and Salzberg 2012) (v2.1.0) with the following settings: --end-to-end, --no-mixed, and -X 2000. Next, all samples were filtered for mapping quality (MAPQ >= 30) and PCR duplicates were marked using Picard tools (Broad Institute 2014), and subsequently removed. Every read was then adjusted for the binding footprint of Tn5 (9 bp) as previously described (Buenrostro et al. 2013); specifically reads were shifted 4 (positive strand) or 5 (negative strand) bp 5' relative to the reference genome. Finally, to give optimal single-base resolution, reads were trimmed to the single 5'-most-base. Then, to assess library quality, we

examined the fragment size distribution of each sample, and observed approximately 147 bp periodicity corresponding to mono-, di-, tri-, etc., nucleosomes, an important indicator of technical sample quality (Buenrostro et al. 2013) (Supplemental Fig. 1A).

ATAC-seq peak calling

For every replicate, prior to calling peaks with MACS (Zhang et al. 2008) (v2.1), single-base reads were shifted 75bp 5' to mimic read distributions of a 150 bp fragment of ChIP-seq, thereby allowing use of MACS. The following settings were used for MACS: -g 9e7, -q 5e-2, --nomodel, --extsize 150, -B, --keep-dup all, and --call-summits. The resulting peaks included a portion that had multiple summits; to maximize our resolution these summits were subsequently separated into individual peaks by treating the midpoint between the two adjacent summits as the 5' and 3' end of each individual peak, respectively.

To identify only the most high-confidence set of peaks, peaks were called for each individual experimental replicate, as well as for pooled replicates from each stage (i.e. all single base pair reads for that stage, regardless of biological replicate), and for two pseudoreplicates which were generated by randomly splitting the pooled samples in half. Consensus peaks for each stage were then classified as any peaks called in the pooled-replicate sample that were at least 50% overlapping A) all biological replicates or B) both pseudoreplicates and at least 2 of the 3 biological replicates (Supplemental Table 2). We used this conservative approach for all our peak-calling analyses except for the transcription factor ChIP-seq intersection analysis in which we used the standard approach of peak calling from pooled replicates.

To account for any unknown issues arising from either biological biases (e.g. Tn5 motif preferences, or undocumented repeats in the *C. elegans* genome), or biases within our analysis

pipeline, we treated our input control, purified *C. elegans* genomic DNA (gDNA) which was transposed with Tn5, in a manner identical to experimental samples through the stage of calling peaks. We expected that reads from the gDNA controls would be uniformly distributed across the genome, and in large part that is what we observed, but unexpectedly we identified regions that had significant pileups of reads. These gDNA pileups highly overlapped annotated repeats (Supplemental Fig. 1B), and reads within the pileups tended to have consistent single nucleotide polymorphisms that were not observed in experimental samples (data not shown). These two pieces of evidence suggest that these peaks arise from PCR artifacts or mapping errors. We took the conservative approach of eliminating consensus peaks that overlapped the gDNA pileups by 20% or more. In addition, we also removed any consensus ATAC-seq peaks that overlapped a blacklist region (a set of regions identified by modENCODE that have "anomalous, unstructured, high signal/read counts in next gen sequencing experiments independent of cell line and type of experiment" (Boyle et al. 2014)) by a single base pair.

A set of 30,832 consensus peaks was generated by first pooling all experimental reads regardless of stage and then calling and masking peaks as described above. This set of peaks was then combined with all stage-specific consensus peaks. To avoid duplicate peaks, overlapping peaks were merged into a single peak, if their summits were within 300 bp. The resulting peak set should include all stage-specific peaks as well as peaks which were not accessible enough to be detected in a single stage, but consistent enough to be detected with all stage-pooled samples (Supplemental Table 3).

Down-sampling

To assess the contribution of sequencing depth differences to the varying number of differentially accessible ATAC-seq peaks, we down sampled each early embryo replicate to 10 million reads. This was done by randomly selecting 10 million inserts for each replicate, and then repeating the consensus peak calling, and subsequent differential accessibility analysis.

Generation of ATAC-seq enrichment scores for mapping

The ATAC-seq single base pair reads for each developmental stage were pooled and quantified at every base in the genome using Bedtools (coverageBed –d); the result being counts of single base pair ATAC-seq reads at every base. This was repeated for the gDNA input control as well. Each sample was then normalized for total sequencing depth to generate the number of reads per million mapped (RPMM) at every base. Finally, enrichment over background was calculated for each developmental stage by taking the log2 of the experimental RPMM divided by the input control RPMM plus 0.1 to avoid 0 at every base in the genome. For display purposes, these enrichment values were binned into 10, 25, or 50 bp non-overlapping windows, the mean enrichment score for that region reported, and the value returned to linear scaling; all values below 0 (i.e. more input signal than experimental signal) were trimmed to 0. All signal sample plots were generated using the R package gViz (Hahne and Ivanek 2016).

Differential accessibility and batch effect removal

To identify regions of dynamic accessibility during development, DiffBind (Stark and Brown 2011) was performed using the consensus ATAC-seq peaks, along with single base pair reads for each biological replicate. In addition, the gDNA control single base pair reads were included along with the score setting of DBA_SCORE_RPKM_FOLD during the counting step, thereby

calculating the fold change between sample and control, after normalizing for sequencing depth. Other non-default settings at this step included, setting bRemoveDuplicates to false, as we had already removed duplicates, setting the fragment size to 1 to avoid any read shifting, and bScaleControl to true. Next, to remove technical variation between biological replicates, we extracted the score calculated by DiffBind and used ComBat (Leek JT 2015). The batch-corrected scores were then returned to DiffBind and differential peak calling completed using the following non-default settings for dba.analyze: bTagwise=FALSE, bFullLibrarySize=TRUE, method=DBA_EDGER, bSubControl=TRUE, bReduceObjects=FALSE. A final FDR of 0.05 was used to call significantly different peaks.

Single replicate ATAC-seq signal correlation

The ComBat normalized signal data for each replicate, which was total enrichment over input in all consensus ATAC-seq peaks, was clustered with the pvclust (Suzuki 2014), using Spearman correlation and the hclust.method = 'complete'. The same Spearman rho values were plotted using the R package pheatmap (Kolde 2013).

ATAC peak enrichment

To assess enrichment of a set of peaks in chromatin states, the portion of peaks overlapping the loci of interest (e.g. promoters) by at least 50% was compared to the median portion of the null distribution meeting the same requirements; the same process was used for transcription factor ChIP-seq peaks, but only a single base pair of overlap was required as not all TF bind in the center of accessibility (Buenrostro et al. 2013). In each case, the null distribution was generated by shuffling the ATAC-seq peaks across the mappable genome (described above) masked for

blacklisted regions (described above) and gDNA peaks (see ATAC-seq peak calling), 10,000 times using Bedtools. The log2 fold enrichment is a comparison between the portion of experimental peaks overlapping features of interest versus the median portion of null distribution peaks overlapping the same features. Significance was assessed by calculating an empirical cumulative distribution function for the null distribution values, and finding the quantile for the experimental peak portion.

ChIP-seq analysis

Histone modifications: The ce10 alignment files for early embryo and larval stage 3 were downloaded from modENCODE (Ho et al. 2014). Reads for young adult samples were downloaded from the modENCODE DCC and aligned to ce10 with BWA (Li and Durbin 2009) (v0.7.9a-r786) using default settings to maintain consistency with the larval and embryo samples. Subsequently, all embryo, larval and young adult samples were filtered for mapping quality (MAPQ >= 30) and duplicates were marked with Picard and removed. Biological replicates were pooled for each histone mark and input, and pseudoreplicates created as described above for ATAC-seq samples. Next, fragment size was estimated via SPP (Kharchenko et al. 2008) for each biological replicate as well as the pooled samples and provided to MACS as "-shiftsize" for peak calling. In addition, the following settings were used for peak calling: -g 9e7, -p 1e-2, --nomodel, -B, --SPMR. Following that, consensus peaks were determined as above for ATAC-seq peaks, but requiring overlap with A) both biological replicates or B) both pseudoreplicates and at least 1 of 2 biological replicates.

Transcription Factors: For early embryo, larval stage 3, and young adult transcription factor binding peaks were taken from Araya, *et al.* 2014(Araya et al. 2014). Only the highest-

confidence ChIP-seqs were used. A complete list of all TFs used is included in Supplemental Table 14.

Chromatin state prediction

Chromatin state predictions were generated using ChromHMM (Ernst and Kellis 2012) (v1.10). The model was built using H3K27ac, H3K27me3, H3K4me1 and H3K36me3 from early embryo, larval stage 3, and young adult samples as well as H3K4me3, H3K9me3, H3K79me2, and H4K20me1 from early embryo and larval stage 3. To maximize the amount of data used to train the model, we also used H3K79me3 in lieu of H3K79me2 in young adult, where H3K79me2 was not available. During the binerization of the genome, settings included: bin = 100bp, p = 1e-3. For prediction of states, a 19-state model was empirically determined to most closely represent the expected states; for example, promoter-like states found near TSSs and transcription-like states found within gene bodies. The emission heatmap created by ChromHMM is presented in Supplemental Fig. 3A. For ease of interpretation, similar states were subsequently merged, resulting in 7 main states: transcribed gene body, promoter/TSS, active enhancer, repressed enhancer, H3K27me3-repressed, heterochromatin, and low signal.

In the process of completing this study, a similar model using a subset of this data, a different program, and data from other organisms was published (Ho et al. 2014). Our models are similar for both early embryo and larval stage 3 (Supplemental Fig. 3D, 3E). Because our model used all the chromosomes, only *C. elegans* data, and included young adults, we have used our model exclusively. However, given the similarity between the models, we would expect similar results with the published model (Supplemental Fig. 3D, 3E).

Motif discovery in ATAC-seq peaks of differential accessibility

de novo motifs were identified using the findMotifsGenome command in Homer (Heinz et al. 2010) (4.7.2). The background for this analysis was all consensus ATAC-seq peaks as these were the all regions considered when calling peaks of differential accessibility. Motif sizes were limited to 6, 8, 10, or 12bp via “-len”, and *de novo* motifs compared to all motifs in the Homer database. Other non-default settings were: -size given -bits. By default, Homer compares the *de novo* motifs to known TF motifs. We report only the TF family as almost all the known motifs were outside of *C. elegans.*

To identify known *C. elegans* motifs, all experimentally derived motifs from cisBP (v1.02) (Weirauch et al. 2014) were provided as known motifs (via “-mknown”) to findMotifsGenome in Homer, using a stringent log odds score of 9 for every motif. This score controls the stringency with which motif matches are made, a lower score allows for degenerate motifs to count as matches, and we found 9 to empirically balance accuracy and stringency.

Predicting accessibility changes with motifs

To predict changes in accessibility between early embryo and larval stage 3, as determined with DiffBind (see above), the number of each mapped *C. elegans* motifs from cisBP (see above) was counted to create a matrix of 166 motif-counts and 30,832 ATAC-seq peaks. The ATAC-seq peaks were then split into two groups: a training set (70%) which the subsequent model was built upon, and a testing-set (30% of all ATAC-seq peaks) which was used to verify the accuracy of the model. We next tried several different classification models to predict whether peaks A) lost accessibility between early embryo and L3, B) stayed consistent between the stages, or C) gained accessibility during development. We found that a generalized boosting model (GBM) (https://CRAN.R-project.org/package=gbm) performed the best, while still allowing for

interpretation of which motifs were the most informative. Given the unbalanced classification problem (~60% of peaks were unchanged, and approximately 20% gained and lost accessibility, respectively), we used balanced accuracy (average of sensitivity and specificity, or the average accuracy of predicting dynamic peaks and static peaks) as our primary metric of classification success. We accurately predicted more than 41.6% of the peaks that increased in accessibility between early embryo and L3. This conservative, yet accurate approach resulted in a balanced accuracy of approximately 0.7 (balanced accuracy is more appropriate for the unbalanced nature of these samples, and has an expected value of 0.5) (Supplemental Fig. 6D). Parameters for the GBM were optimized using the R package, caret (Kuhn 2015), to run 10-fold cross validation; the following parameters were optimized: interaction depth (how many levels there are in the individual trees): 2, 5, 8 or 11 and the number of trees to use per model: 6,000, 10,000, or 14,000. Otherwise default settings were used. Using the fit or prediction from this model every peak in each set, training and testing, respectively, was split into 3 classes, decreased accessibility during development, consistent, or increased accessibility.

An important aspect of the model selected is that it allows for interpretation of which motifs were the most influential or important in predicting the changes in accessibility. These values are arbitrary, but relative within the model (i.e. a motif with a score of 10 was twice as informative in predicting chromatin accessibility changes as a motif with a score of 5).

RNA-seq analysis

Reads were downloaded from the modEncode DCC, and then processed with trimGalore (v0.2.3) (Krueger 2015), where both stringency and quality were set at 15. Alignment to the RefSeq transcriptome and the ce10 genome, including mitochondrial sequence, was completed with

Tophat (v2.0.9) (Kim et al. 2013). For all samples the following settings were used: -g 10, --no-coverage-search, --no-novel-indels, and --no-novel-juncs --b2-very-sensitive. For 36 bp reads, segment-length was set to 17, while 76 bp reads used the default segment-length setting. Quantification of reads over RefSeq genes was performed with HTSeq (v0.6.1) (Anders et al. 2015). Differential genes were identified with edgeR (v3.8.5) (Robinson et al. 2010). Refseq genes were subsequently converted to Ensembl names for comparison to ATAC-seq.

GRO-seq analysis

Raw data from (Kruesi et al. 2013) was aligned to the entire ce10 genome, including the mitochondrial genome using bowtie2 and the --very-sensitive settings. Quantification of FPKM in RefSeq genes was completed with Homer (v4.7.2) (Heinz et al. 2010)

Conservation calculations

As a measure of conservation, we downloaded the PhastCons 7-way track from the UCSC Genome browser (Rosenbloom et al. 2015). This track assigns a score at the single base pair level using the conservation between seven nematode species. The score ranges from 0 to 1, where a higher score indicates better conservation. To avoid biases, unmappable regions (described above), all blacklisted regions (described above), and all ATAC-seq gDNA peaks were excluded from further analysis. In addition, Ensembl protein-coding exons (Rosenbloom et al. 2015) were excluded from further analysis to focus on non-coding conservation, a hallmark of regulatory regions. To focus on distal regulatory regions, we also excluded all regions 1 kb upstream and 0.5 kb downstream of TSSs. The remaining portion of the genome we refer to as the distal non-coding genome.

To calculate a conservation score for a set of regions (e.g. ATAC-seq peaks), the median single base pair conservation score was calculated for every region in which at least half of the region was included in the distal non-coding genome described above. To assess the significance of any such result, we generated a null distribution for every set of regions. To do this, we selected only those regions at least half within the distal non-coding genome, and we randomized the location of the peaks requiring that they were at least half within the distal non-coding genome. We repeated this 10,000 times and calculated the single base pair median phastCons score for each region every time.

Chromatin state changes between stages

To identify which transitions in ChromHMM-predicted chromatin states that our dynamic ATAC-seq peaks were enriched for, we split consensus ATAC-seq peaks into 3 classes: significantly more accessible in EE, significantly more accessible in L3 and unchanged. For each set, we annotated each peaks' early embryo ChromHMM predicted chromatin state by requiring at least 50% of the ATAC-seq peak to overlap with the state. This split each of the 3 sets of ATAC-seq peaks into 7 classes (one for each chromatin state). Then for each of those classes in each set, we annotated the L3 ChromHMM-predicted chromatin state as above. The result of this process was a 7x7 matrix corresponding to the 7 chromatin states for each set of ATAC-seq peaks. These were converted to portions in a row-wise manner (i.e. each row summed to 1), and then log2 enrichments were calculated for the dynamic ATAC-seq peaks versus the consistent ATAC-seq peaks.

GO term enrichment

To identify the underlying biological process marked by chromatin accessibility dynamics, every ATAC-seq peak was associated with the nearest TSS, up to 10kb away. Genes were then ordered by the total change in ATAC-seq signal in their associated ATAC-seq peaks between the two stages being compared (e.g. early embryo versus larval stage 3). This approach thus considers both the number of ATAC-seq peaks and the intensity of signal in the peaks associated with genes. Gene Ontology enrichments were then calculated with the online GO program, GOrilla (Eden et al. 2009), using the single ranked list setting, and the fast mode was not used to optimize accuracy.

Heatmap plots

All read-based heatmaps were generated with NGS Plot (Shen et al. 2014) with default settings except for the color distribution (-CD), which was set to 1, thereby centering the color scale on 0. For ATAC-seq, pooled-replicate BAM (alignment) files of single base pair insert sites for each experimental stage versus the input control were used; fragment size was empirically set at 25bp. For histone modification ChIP-seq, pooled-replicate BAM files of the reads and input controls were used.

Insert size calculation

Calculations and plots were generated with Picard Tools from the quality-filtered aligned ATAC-seq reads.

Calculation of genomic loci overlap

The Jaccard index was calculated using Bedtools Jaccard; specifically: (length of intersection of 2 sets of genomic loci (bp)) / ((length of union) – (length of intersection)).

REFERENCES

Anders S, Pyl PT, Huber W. 2015. HTSeq--a Python framework to work with high-throughput sequencing data. *Bioinformatics* **31**(2): 166-169.

Araya CL, Kawli T, Kundaje A, Jiang L, Wu B, Vafeados D, Terrell R, Weissdepp P, Gevirtzman L, Mace D et al. 2014. Regulatory analysis of the C. elegans genome with spatiotemporal resolution. *Nature* **512**(7515): 400-405.

Boyle AP, Araya CL, Brdlik C, Cayting P, Cheng C, Cheng Y, Gardner K, Hillier LW, Janette J, Jiang L et al. 2014. Comparative analysis of regulatory information and circuits across distant species. *Nature* **512**(7515): 453-456.

Broad Institute. 2014. Picard Tools.

Buenrostro JD, Giresi PG, Zaba LC, Chang HY, Greenleaf WJ. 2013. Transposition of native chromatin for fast and sensitive epigenomic profiling of open chromatin, DNA-binding proteins and nucleosome position. *Nature methods* **10**(12): 1213-1218.

Chen RA, Down TA, Stempor P, Chen QB, Egelhofer TA, Hillier LW, Jeffers TE, Ahringer J. 2013. The landscape of RNA polymerase II transcription initiation in C. elegans reveals promoter and enhancer architectures. *Genome research* **23**(8): 1339-1347.

Eden E, Navon R, Steinfeld I, Lipson D, Yakhini Z. 2009. GOrilla: a tool for discovery and visualization of enriched GO terms in ranked gene lists. *BMC bioinformatics* **10**: 48.

Ernst J, Kellis M. 2012. ChromHMM: automating chromatin-state discovery and characterization. *Nature methods* **9**(3): 215-216.

Haenni S, Ji Z, Hoque M, Rust N, Sharpe H, Eberhard R, Browne C, Hengartner MO, Mellor J, Tian B et al. 2012. Analysis of C. elegans intestinal gene expression and polyadenylation by fluorescence-activated nuclei sorting and 3'-end-seq. *Nucleic acids research* **40**(13): 6304-6318.

Hahne F, Ivanek R. 2016. Visualizing Genomic Data Using Gviz and Bioconductor. *Methods in molecular biology* **1418**: 335-351.

Heinz S, Benner C, Spann N, Bertolino E, Lin YC, Laslo P, Cheng JX, Murre C, Singh H, Glass CK. 2010. Simple combinations of lineage-determining transcription factors prime cis-regulatory elements required for macrophage and B cell identities. *Molecular cell* **38**(4): 576-589.

Ho JW, Jung YL, Liu T, Alver BH, Lee S, Ikegami K, Sohn KA, Minoda A, Tolstorukov MY, Appert A et al. 2014. Comparative analysis of metazoan chromatin organization. *Nature* **512**(7515): 449-452.

John S, Sabo PJ, Thurman RE, Sung MH, Biddie SC, Johnson TA, Hager GL, Stamatoyannopoulos JA. 2011. Chromatin accessibility pre-determines glucocorticoid receptor binding patterns. *Nature genetics* **43**(3): 264-268.

Kharchenko PV, Tolstorukov MY, Park PJ. 2008. Design and analysis of ChIP-seq experiments for DNA-binding proteins. *Nature biotechnology* **26**(12): 1351-1359.

Kim D, Pertea G, Trapnell C, Pimentel H, Kelley R, Salzberg SL. 2013. TopHat2: accurate alignment of transcriptomes in the presence of insertions, deletions and gene fusions. *Genome biology* **14**(4): R36.

Kolde R. 2013. pheatmap: Pretty Heatmaps.

Krueger F. 2015. TrimGalore.

Kruesi WS, Core LJ, Waters CT, Lis JT, Meyer BJ. 2013. Condensin controls recruitment of RNA polymerase II to achieve nematode X-chromosome dosage compensation. *eLife* **2**: e00808.

Kuhn M, Contributions from Jed Wing and Steve Weston and Andre Williams and Chris Keefer and Allan Engelhardt and Tony Cooper and Zachary Mayer and Brenton Kenkel and the R Core Team and Michael Benesty and Reynald Lescarbeau and Andrew Ziem and Luca Scrucca,. 2015. caret: Classification and Regression Training.

Langmead B, Salzberg SL. 2012. Fast gapped-read alignment with Bowtie 2. *Nature methods* **9**(4): 357-359.

Leek JT JW, Parker HS, Fertig EJ, Jaffe AE and Storey JD. 2015. sva: Surrogate Variable Analysis.

Li H, Durbin R. 2009. Fast and accurate short read alignment with Burrows-Wheeler transform. *Bioinformatics* **25**(14): 1754-1760.

Mello C, Fire A. 1995. DNA transformation. *Methods in cell biology* **48**: 451-482.

Quinlan AR, Hall IM. 2010. BEDTools: a flexible suite of utilities for comparing genomic features. *Bioinformatics* **26**(6): 841-842.

R Core Team. 2013. R: A Language and Environment for Statistical Computing. R Foundation for Statistical Computing, Vienna, Austria.

Weirauch MT, Yang A, Albu M, Cote AG, Montenegro-Montero A, Drewe P, Najafabadi HS, Lambert SA, Mann I, Cook K, et al. 2014. Determination and inference of eukaryotic transcription factor sequence specificity. *Cell* **158**(6):1431-43

Robinson MD, McCarthy DJ, Smyth GK. 2010. edgeR: a Bioconductor package for differential expression analysis of digital gene expression data. *Bioinformatics* **26**(1): 139-140.

Rosenbloom KR, Armstrong J, Barber GP, Casper J, Clawson H, Diekhans M, Dreszer TR, Fujita PA, Guruvadoo L, Haeussler M et al. 2015. The UCSC Genome Browser database: 2015 update. *Nucleic acids research* **43**(Database issue): D670-681.

Shen L, Shao N, Liu X, Nestler E. 2014. ngs.plot: Quick mining and visualization of next-generation sequencing data by integrating genomic databases. *BMC genomics* **15**: 284.

Stark R, Brown, G 2011. DiffBind: differential binding analysis of ChIP-Seq peak data.

Suzuki R, Shimodaira, Hidetoshi 2014. pvclust: Hierarchical Clustering with P-Values via Multiscale Bootstrap Resampling.

Zhang Y, Liu T, Meyer CA, Eeckhoute J, Johnson DS, Bernstein BE, Nusbaum C, Myers RM, Brown M, Li W et al. 2008. Model-based analysis of ChIP-Seq (MACS). *Genome biology* **9**(9): R137.
