# Supplementary material for: Chromatin accessibility dynamics reveal novel functional enhancers in C. elegans
Source: Genome Res. 2017 Dec;27(12):2096–107. doi: 10.1101/gr.226233.117 (PMC5741055; doi:10.1101/gr.226233.117)
Supplement: Supplemental Material [file supp_gr.226233.117_Supplemental_Extended_Protocol.docx]

*C. elegans* ATAC-seq

Aaron C. Daugherty, PhD

Written: December 8^th^, 2014; Updated May 14^th^, 2017

This whole-*C. elegans* ATAC-seq protocol was developed for “Chromatin accessibility dynamics reveal novel functional enhancers in *C. elegans*)” (Daugherty *et al*.). For this study, we grew worms on standard solid media and used tight temporal synchronization (45min) achieved without bleach. These steps were performed to ensure that environmental effects did not alter chromatin signatures while minimizing sample variation.

**Protocol outline**:

1. For each replicate, grow1-2 10cm plates of well-synced C. elegans aged to the appropriate point
2. Harvest nuclei
3. Perform standard ATAC protocol
4. QC libraries
5. Sequence

**Reagents and tools:**

1. 2x NPB (see attached Excel sheet)
2. Wheaton stainless steel tissue homogenizer: http://www.amazon.com/Wheaton-Stainless-Dura-Grind-Grinder-Overall/dp/B00797LTQG  (clearance 0.0005 inches

=

12.5

µm). I use the small one (7mL), and I clean it with: H2O, bleach, EtOH, milliPore H2O
3. Nextera DNA Sample Preparation Kit
4. NEBNext High-Fidelity 2X PCR Master Mix
5. Qiagen mini elute columns
6. Pure SYBR (not a master mix)
7. 1.5mL Non-stick microfuge tubes
8. Standard lab stocks

**Detailed protocol**

Everything here is for a single replicate, though I tend to grow an excess number of worms for ease.

1. **Growing well-synced worms**
   1. Bleach a single mixed stage 6cm plate, and split to 3-4 10cm plates seeded with ~500uL of 2x OP50
      1. Avoid starving due to possible transgenerational inheritance issues changing chromatin signature
   2. Grow at 20C for ~2.5days
   3. Once gravid, wash off all plates with M9 into a 15mL conical tube.
      1. *By harvesting worms as they are starting to lay eggs, and before L1s have hatched, subsequent syncing will be improved.*
   4. Wash 1x with M9.
   5. If growing larval or adult stages:
      1. Split the worms evenly between 3-4 fresh 10cm plates seeded as above.
         1. *After the last wash, remove the SN to ~<200µL to avoid putting too much liquid on the plates*
      2. After the plates are dry (should be a few minutes at most), return to 20°C for 45min
      3. Wash off all plates.
         1. *If generating gDNA input controls, use these parents (see below), otherwise discard.*
      4. Approximately every hour check for and remove any remaining parents
         1. *One or two will occasionally ‘hide’ on the edge of the plate*
      5. Grow worms to appropriate age
      6. Harvest by washing off with M9
      7. Wash >= 2x with M9
      8. Flash freeze in liquid N2, store at -80°C
   6. If harvesting early embryos:
      1. Do standard beach prep (see Wormbook), being sure to remove all parental worm parts
      2. Wash 2X with M9
      3. Flash freeze in liquid N2, store at -80°C until ready to complete steps 2 and 3 together
2. **Harvesting nuclei**

*This is adapted from the FANS paper: <http://www.ncbi.nlm.nih.gov/pubmed/22467213>

- Using non-stick tubes helps with yield.

- 1. Before you start:
     1. Chill Wheaton at 4°C
        1. *Do NOT chill at -20*°*C*
     2. Make 4x NPB- (see below)
     3. Immediately before beginning, add fresh reagents, to final concentration of 2X
     4. Pre-chill centrifuge to 4°C
  2. Thaw worm pellet (from above) on ice and perform all subsequent steps on ice.
  3. Transfer worms to a pre-chilled Wheaton stainless-steel tissue grinder. Add an equal volume of ice-cold 2x NPB (see below and attached Excel sheet)
     1. *Ensure the final volume is at least ~150µL or the Wheaton will not work well*
  4. Homogenize by doing 2 to 3 strokes but no more than 4
     1. *More may burst the nuclei*
  5. Let the unbroken worms and big worm pieces settle to the bottom of the Wheaton
     1. *Approximately 5 minutes is generally enough*
  6. Separate supernatant (SN) (contains nuclei) from pellet by spinning at lowest possible speed (200g) in chilled centrifuge
  7. Transfer SN to fresh tube on ice
  8. Resuspend pellet in NPB 2x
     1. *Again at least a total volume of ~150µL*
  9. Repeat steps d-h until there are no visible worm pieces, each time adding the SN to the same tube
     1. *After the last sample wash out the Wheaton with extra 2xNPB*
  10. After pooling the SN samples, perform another 200g spin and transfer the SN to a final tube
      1. *Optional, but helpful*
  11. To pellet the nuclei, spin at 1000g for 10min
  12. Once the nuclei are pelleted, COMPLETELY remove the SN
      1. *The nuclei will not be visible, so make sure to note where the pellet should be – usually by placing the tube tab up before spinning*
      2. *Leaving even a few µL can result in sequencing a lot bacterial DNA if you’re working with larval or adult stages.*
  13. Go directly to step 3
  14. If performing input control/gDNA prep:
      1. Using embryos will avoid any bacterial contamination issues
      2. Isolate gDNA from nuclei (standard approach):
         1. proteinaseK
         2. RNase
         3. Phenol/chloroform extract
      3. Rather than using nuclei in step 3b (below), add 10ng of very clean gDNA. Otherwise treat it as other samples

1. **ATAC**
   1. While pelleting nuclei above, prepare ATAC buffer: 25µL 2X tagmentation buffer, 22.5µL H2O
   2. Resuspend the pelleted nuclei with 47.5µL of buffer from step 3a.
   3. Add 2.5µL of tagmentation enzyme (i.e. Tn5)
   4. Incubate at 37°C for 30mins, then move to 4°C
      1. *If performing several preps in sequence, let samples sit at 4°C for up to a few hours (longer may be okay), while completing subsequent samples.*
   5. Perform a standard Qiagen mini-elute column clean up
      1. The only modification is that following the PE wash and subsequent spin, carefully suck off any droplets of remaining EtOH from the rubber gasket in the column
         1. *Be careful not to puncture the filter*
         2. *This is because even 1µL of EtOH can be problematic when you’re eluting in 10µL*
      2. Elute in 10µL of warm EB
   6. Samples can be frozen at -20°C until all preps are ready
   7. Perform PCR amplification
      1. Wait until all samples are ready
      2. Set up a 50µL reaction using all 10µL of a sample as template
         1. See attached Excel sheet for details
         2. Assuming you’re multiplexing, each reaction should get a unique primer2 (The [supplement](http://www.nature.com/nmeth/journal/v10/n12/full/nmeth.2688.html#supplementary-information) of the original paper lists all of the primers you might need; we ordered ours as we would any other primer)
   8. Perform the first 5 cycles, but then hold the reactions at 4°C
   9. While those 5 cycles are running, set up the qPCR reactions
      1. See Excel file for details
      2. Which primer 2 you use doesn’t matter because they’re all similar enough to work
   10. Use 5µL of the PCR from step h as template for the qPCR reaction
       1. Use the same thermocycling steps as for step h
   11. Once each sample in the qPCR has plateaued, find the number of cycles it took for each to reach the 1/3max fluorescence value
       1. The idea is to make sure you amplify your samples to the beginning of the linear portion of the graph, so that you do not overamplify your libraries
   12. Return to the original PCR in step h and let each sample continue the number of cycles noted in step k.
   13. Once all are done, perform a standard Qiagen PCR clean up, elute in 25µL of warm water (or dilute TE), let stand for a few minutes to get optimal yield.
2. **Quality Control (QC) libraries & sequence**
   1. Run ~3µL of sample on a 5% TBE gel (we use precast from biorad).
      1. You’re looking for laddering of around 150bp which indicates that you have reads corresponding to mono-, di-, tri-, etc nucleosomes
      2. This step can be skipped, but if you’re just starting it’s a good idea to perform.
         1. *If you don’t see laddering, you may want to further amplify your samples for QC, but do sequencing those libraries is not recommended as they will be overamplified*
   2. Submit a few µL to the bioAnalyzer, interrogating the fragments b/t 100 and 700bp.
      1. *There is a good chance that you will have larger fragments as well, and these are most likely gDNA flow through that will not stick to the sequencing chip, but they do complicate quantifying total DNA.*
      2. *More accurate quantification can be performed using a qPCR based approach, see the original ATAC-seq paper.*
   3. Submit to the sequencer using the concentration you got from step b.
      1. These are odd looking libraries and sequencing facilities may be surprised/concerned if they are expecting ChIP-seq looking libraries. These libraries need to be clustered more tightly (i.e. put on more DNA than you normally would for a ChIP-seq library) and that’s because there is a portion of the DNA that is in fact not sequence-able because it doesn’t have the adaptors on it (the gDNA flow through from above).
      2. If your facility has not worked with ATAC-seq samples before, it may be worthwhile to try an experimental run with samples at a range of concentrations.
